# Performance of Hearing Test Software Applications to Detect Hearing Loss

**DOI:** 10.1001/jamanetworkopen.2025.2166

**Published:** 2025-03-27

**Authors:** Meaghan Lunney, Natasha Wiebe, Tanis Howarth, Lorienne Jenstad, Alex DeBusschere, Gillian Crysdale, Sharon Straus, Kara Schick-Makaroff, Maoliosa Donald, Stephanie Thompson, Jayna Holroyd-Leduc, Marcello Tonelli

**Affiliations:** 1 Department of Medicine, University of Calgary, Calgary, Canada; 2 Department of Medicine, University of Alberta, Edmonton, Canada; 3 Alberta Health Services, Calgary, Canada; 4 School of Audiology and Speech Sciences, University of British Columbia, Vancouver, Canada; 5 Department of Medicine, University of Toronto, Toronto, Canada; 6 Faculty of Nursing, University of Alberta, Edmonton, Canada

## Abstract

**Importance:**

Hearing loss is common and may impact health and quality of life if not properly managed. It is diagnosed following formal audiological assessment, which may not be available or practical. Hearing test software applications (apps) may help identify people who might benefit from audiological assessment, but their diagnostic accuracy has been incompletely studied.

**Objective:**

To measure and compare the validity and reliability of 2 commonly recommended apps (hearWHO and SHOEBOX) to detect moderately severe or greater hearing loss. Secondary objectives were to evaluate the apps’ ability to detect less severe hearing loss and the diagnostic performance of 2 questionnaires for detecting both severities of hearing loss.

**Design, Setting, and Participants:**

This prospective diagnostic accuracy study compared the hearWHO and SHOEBOX apps with a 4-frequency pure-tone average audiological assessment reference standard. All consenting English-speaking patients aged 18 years or older and referred for routine audiological assessment at a publicly funded health center in Calgary, Canada, were included between May 17, 2023, and March 12, 2024.

**Main Outcome and Measures:**

The main outcome was the validity and reliability of 4 index tests, including the hearWHO app, SHOEBOX app, Revised Hearing Handicap Inventory–Screening (RHHI-S) questionnaire, and the Single-Item Self-Assessment (SISA) questionnaire, to detect moderate to severe hearing loss. All index test results were compared with an audiological assessment reference standard (hearing loss defined by a better ear hearing threshold of ≥50 dB [more severe denoted as HL_50_] or ≥20 dB [less severe denoted as HL_20_]). Test-retest reliability of the 2 apps and C statistics, sensitivity, specificity, and positive and negative predicted values of all index tests were measured.

**Results:**

A total of 130 participants were recruited (median [IQR] age, 58 [47-67] years; 82 female [63.1%]). Complete data for each comparison ranged from 123 to 129 participants. The prevalence of HL_50_ was 16.3% (21 or 130 participants). Neither the hearWHO nor the SHOEBOX app had high test-retest reliability (all κ-values <0.80), with the SHOEBOX having a κ of 0.64 (95% CI, 0.48-0.79) and hearWHO having a κ of 0.32 (95% CI, 0.18-0.46). All C statistics for HL_50_ were less than 0.80. When testing for HL_50_, diagnostic performance for both apps was better for the second measurement than the first measurement or the mean. Sensitivity and specificity for the second measurement of SHOEBOX were 0.26 (95% CI, 0.09-0.51) and 1.00 (95% CI, 0.97-1.00), respectively, and for the second measurement of hearWHO, 0.67 (95% CI, 0.43-0.85) and 0.71 (95% CI, 0.62-0.79), respectively. Sensitivity and specificity for the RHHI-S were 0.76 (95% CI, 0.53-0.92) and 0.42 (95% CI, 0.32-0.52), respectively, and for SISA, 0.10 (95% CI, 0.01-0.30) and 0.90 (95% CI, 0.83-0.95), respectively. Using a less stringent diagnostic threshold with SHOEBOX increased sensitivity for HL_50_ to at least 95% while retaining a specificity of 47% to 54%. Sensitivity and specificity for both apps were higher for HL_20_.

**Conclusions and Relevance:**

These findings suggest that both hearWHO and SHOEBOX have limited test-retest reliability, perhaps because of a learning effect. Both apps may be suitable if a sensitive strategy is desired for identifying people who may benefit from diagnostic audiological assessment, whereas the SHOEBOX app may be preferable if a specific strategy is desired. If neither app is available, the RHHI-S or the SISA could be used depending on whether sensitivity or specificity is desired.

## Key Points

**Question:**

What is the validity and reliability of 2 commonly recommended hearing loss mobile applications (apps), hearWHO and SHOEBOX, in detecting moderate to severe hearing loss?

**Findings:**

In this diagnostic study of 130 participants (21 [16.3%] with hearing loss), sensitivity and specificity of the SHOEBOX and hearWHO apps to detect moderate to severe hearing loss were slightly better than the Revised Hearing Handicap Inventory–Screening and the Single-Item Self-Assessment questionnaires compared with the audiological assessment reference standard. Sensitivity and specificity for the SHOEBOX app were higher for mild hearing loss.

**Meaning:**

These findings suggest that both the SHOEBOX and hearWHO apps may be suitable if a sensitive strategy is desired for identifying people who may benefit from diagnostic audiological assessment, whereas the SHOEBOX app may be preferable if a specific strategy is desired.

## Teaser

This diagnostic study compares the validity and reliability of the hearWHO and SHOEBOX mobile applications with the audiological assessment reference standard for detecting moderate to severe hearing loss in adult patients.

## Introduction

Hearing loss affects 1 in every 5 people^1^ and can have severe physical, social, cognitive, economic, and emotional consequences, as well as lead to reduced quality of life.^2^ People with moderately severe hearing loss or greater (a hearing threshold of ≥50 dB in their better ear) may experience difficulty hearing conversational speech, even in quiet environments.^3^ Hearing loss is associated with poor cognitive function, social relationships, and mental health,^4^ as well as a higher risk of falls, cardiovascular events, dementia, long-term-care placement, emergency department visits, and days in the hospital.^5^

Early identification and management of hearing loss is important to delay progression and reduce the risk of related consequences. Hearing loss is optimally diagnosed using a comprehensive audiological assessment performed by a regulated hearing health care professional. However, this assessment is not always readily available or practical. Hearing test software applications (apps) have been developed to help identify people who may benefit from diagnostic audiological testing and intervention.

There are 2 types of hearing loss detection apps: (1) pure-tone threshold–based and (2) speech-based.^6^ Similar to the reference standard audiogram, pure-tone threshold apps estimate a hearing threshold in decibels hearing level (dB HL) using discrete pure-tone frequency stimuli. In contrast, speech-based apps measure hearing levels using a speech-in-noise procedure.^7^ A commonly used speech-based hearing test is the digits-in-noise test, which presents the listener with recorded digit triplets (eg, 6-2-8) spoken with speech-shaped (ie, background masking) noise.^8^ Digits-in-noise apps measure either the speech recognition threshold or percentage-correct scores.^6^ The hearWHO app is a widely recommended smartphone-based digits-in-noise test developed by the World Health Organization,^9^ validated against telephone-based digits-in-noise tests, and used in more than 200 000 people worldwide.^9,10,11^ The SHOEBOX Online app (SHOEBOX Ltd) combines self-reported measures of hearing loss and pure-tone threshold measurements. Both apps are widely available and are promoted as quick, free, and easy ways to detect people who may have hearing loss. However, limited data have evaluated these apps’ performance against a reference standard measure,^12^ and to our knowledge, no studies have been done to directly compare their performance.

We designed this prospective study to measure and compare the validity and reliability of the hearWHO and SHOEBOX apps to detect moderate to severe hearing loss, representing a group of people in whom hearing loss should be easier to detect and who are very likely to benefit from treatment. We defined this degree of hearing loss by a better ear hearing threshold of 50 dB HL or greater on audiological assessment^3^ (hereafter referred to as HL_50_). Because the optimal strategy depends on the goal of testing, we also evaluated the validity and reliability of the 2 apps to detect less severe hearing loss, as defined by a hearing threshold of 20 dB HL or greater on audiological assessment (hereafter referred to as HL_20_). This degree of hearing loss may be more difficult to detect but may still benefit from treatment if identified. Finally, as alternatives to apps, we assessed the diagnostic performance of 2 instruments: the Revised Hearing Handicap Inventory–Screening (RHHI-S)^13^ and the Single-Item Self-Assessment (SISA)^14^ questionnaires.

## Methods

This prospective diagnostic accuracy study was approved by the Conjoint Health Research Ethics Board at the University of Calgary, and all participants provided written informed consent. The study followed the Standards for Reporting Diagnostic Accuracy (STARD) reporting guideline.^15^

### Participants

We invited all eligible patients undergoing audiological assessment at a single health center in Calgary, Canada, between May 17, 2023, and March 12, 2024, to participate. Adult (aged ≥18 years) English-speaking individuals able to provide informed consent, who resided in Alberta, and who had a valid personal provincial health number were eligible for inclusion. We excluded individuals who had already been diagnosed with hearing loss by prior audiological testing, used hearing aids, had any type of hearing implant, had ear pain or ear drainage, and had recent basal skull trauma or prior temporal bone fracture.

Posters about the study were displayed at the audiology center. At the end of each assessment, audiologists explained the study to each eligible patient and directed them to the onsite research coordinator if they were interested. Consenting participants met with a research coordinator (A.D.) who was trained in the use of the study measures. The coordinator collected demographic and clinical information and instructed the participants on how to self-administer the index hearing tests. The coordinator provided the tablet and headphones. The research coordinator had access to an amplification device to facilitate communication with participants, if required. Index hearing tests were administered immediately following the audiological assessment (reference standard).

### Reference Standard

As used in clinical practice, a 4-frequency pure-tone average was calculated from the full audiological examination completed by registered audiologists.^16^ We measured 4-frequency pure-tone average values and then categorized them as definite, possible, or no hearing loss to align with cutoff values and categories used by the mobile apps.

First, a 4-frequency pure-tone average threshold was determined from measurements at 500, 1000, 2000, and 4000 Hz. If the lowest hearing level obtained was 50 dB HL or greater (corresponding to moderately severe hearing loss), we categorized the participant as having HL_50_. If the lowest hearing level obtained was less than 50 dB HL, a second 4-frequency pure-tone average threshold was calculated from measurements at 3000, 4000, 6000, and 8000 Hz. If this lowest hearing level was 50 dB HL or greater, the participant was also considered to have HL_50_. If the lowest hearing level was 20 dB HL or greater, we categorized the participant as having HL_20_. Participants for whom the lowest hearing level was less than 20 dB HL were categorized as having no hearing loss. We tested each ear separately, but used the result from the better ear for each participant.

### Index Hearing Tests

There were 4 index tests administered: the hearWHO app, the SHOEBOX Online test, the RHHI-S,^13^ and the SISA.^14^ If an index test had only 1 category that referred to an abnormal result, we described that result as definite hearing loss. For the 2 index tests that had 2 categories that referred to an abnormal result (ie, more severe and less severe abnormality in SHOEBOX and SISA), we described the more severe abnormality as definite hearing loss and the less severe abnormality as possible hearing loss.

The hearWHO and SHOEBOX apps were administered using an Apple iPad, 9th generation (Apple Inc) and HD 280 Pro headphones (Sennheiser). The average ambient noise level over 10 seconds in the room was measured using the Decibel X:dB Sound Level Meter app (Apple Inc).^17^

The hearWHO app is a smartphone-based hearing test. Through headphones, the application emits 23 sets of digit triplets at various frequencies using the digits-in-noise methodology. The resulting score ranges from 0 to 100 points. Scores less than 50 points indicate some degree of hearing loss. Scores greater than 75 points are considered good hearing.^18^ Scores between 50 and 75 points are intermediate, and follow-up monitoring is advised. We defined definite hearing loss by a hearWHO result of less than 50 points (hereafter referred to as definite HL_hearWHO_) in the better ear.

The SHOEBOX Online test includes 3 self-assessment questions and 6 threshold-finding stimuli using pure-tone average technology. While each ear was tested separately, we used the best result from both ears. All participants did the SHOEBOX test twice during a single assessment period. The result is given in 3 categories: significant hearing loss, hearing loss, or good hearing.^19^ We defined definite hearing loss by a SHOEBOX result of significant hearing loss (hereafter referred to as definite HL_SHOEBOX_). We defined possible hearing loss by a SHOEBOX result of significant hearing loss or hearing loss (hereafter referred to as HL_SHOEBOX_).

The SISA^14^ consists of a single question, “Do you have any difficulty with your hearing?” There are 4 responses, which are abbreviated as follows: yes, almost always; yes, regularly; yes, sometimes; and no. We defined definite hearing loss by an answer to the SISA of yes, almost always, or yes, regularly (hereafter referred to as definite HL_SISA_) and possible hearing loss by any answer to the SISA except no (hereafter referred to as HL_SISA_).

The RHHI-S is a 10-item questionnaire.^13^ Each question has 3 possible responses: yes (4 points), sometimes (2 points), and no (0 points), meaning that the total possible score ranges from 0 to 40 points. We defined definite hearing loss as a score of at least 6 points (hereafter referred to as HL_RHHI-S_). eTable 1 in Supplement 1 provides further details on the index texts.

### Data Collection

The study population was a convenience series. Participants were first seen and assessed by an audiologist using conventional audiometry. The results of the examination, including the reference standard test, were then shared with the participants in accordance with best practice guidelines. Immediately after the examination, the participants were referred to the onsite research coordinator, who was unaware of the examination results. The participant then completed 2 hearWHO app and 2 SHOEBOX tests in a quiet, but not soundproofed room. The tests were administered in random order. Prior to beginning the first test, the average ambient noise in the room was measured over a period of 10 seconds. The amount of time taken to complete each test was recorded in minutes. Following the tests with the apps, the participants completed the RHHI-S and SISA instruments. The participant was not masked to results from the index tests. When needed, the research coordinator used a Pocketalker Ultra device (Williams Sound) to amplify voices to communicate more effectively with the participants. Demographic characteristics potentially associated with hearing loss (including age, self-identified gender [man, woman, another gender], sex at birth, translator preferred, education, and material deprivation quintile) and medical history were collected from participant interviews and electronic medical records and entered into a Research Electronic Data Capture database (REDCap). Data on race and ethnicity were not collected. The Pampalon Index was used to assess material deprivation.^20^ The index categorizes participants based on their residential postal code into 5 bins of socioeconomic inequalities in health care services and population health, with 5 representing neighborhoods with the most deprivation.

### Sample Size Calculation

There are approximately 150 000 adults with hearing loss living in Alberta. An estimated 25% of adults aged 65 to 74 years have disabling hearing loss.^21^ A sample of 124 participants from a finite population of 150 000, in whom approximately 25% had moderately severe hearing loss, would produce a 2-sided 95% CI with a precision (half-width) of −0.025 to 0.025 when the sample C statistic is 0.800. A sample size of 124 would also produce a 2-sided 95% CI with a precision (half-width) of −0.070 to 0.070 when the actual sensitivity or specificity is near 0.800. These estimates were determined using PASS, version 23.0.5 software (NCSS).

### Statistical Analysis

We did all analyses using Stata/MP, version 18.0 (StataCorp LLC). We summarized demographic and clinical characteristics of the study population as counts and percentages or medians (IQRs), as appropriate. To assess test-retest reliability (for the hearWHO score and 3-category grouping and for the SHOEBOX 3-category grouping), we reported the proportion of agreement and the simple κ statistic. For calibration, we reported the simple κ statistic for each index test against the reference standards for HL_50_ and HL_20_. For discrimination, we reported the C statistic, sensitivity, specificity, positive predictive value, and negative predictive value. We reported 95% CIs for all statistics. In exploratory post hoc analyses, we aimed to find the optimal threshold for diagnosing HL_50_ using the hearWHO score and the RHHI-S score.

## Results

### Participants

We recruited 130 participants (median [IQR] age, 58 [47-67] years; 82 assigned female sex at birth [63.1%], 48 assigned male sex at birth [36.9%]) (Table 1). Sixty-five participants (50.8%) had a university education, 9 (7%) would have preferred a translator, 42 (32.3%) had been hospitalized within the past 5 years, 129 (99.2%) had home internet access, 106 (81.5%) used social media, and 114 (87.7%) used a smartphone.

**Table 1.** Participant Characteristics (N = 130)

Characteristic	Participants, No. (%)
Age, median (IQR), y	58 (47-67)
Gender^a^	
Man	47 (36.7)
Woman	80 (62.5)
Another gender	1 (0.8)
Sex at birth	
Female	82 (63.1)
Male	48 (36.9)
Translator preferred^a^	9 (7.0)
Education^a^	
Less than high school	2 (1.6)
High school graduate	8 (6.2)
High school graduate certificate and/or some post secondary	28 (21.9)
College certificate and/or diploma	25 (19.5)
University certificate, diploma, or degree	65 (50.8)
Material deprivation^a^	
1 (Least)	42 (33.1)
2	33 (26.0)
3	29 (22.8)
4	16 (12.6)
5 (Most)	7 (5.5)
Prior hospitalization	42 (32.3)
Social media use	106 (81.5)
Computer use	113 (86.9)
Smartphone use	114 (87.7)
Cellphone use	38 (29.2)
Tablet use	68 (52.3)
Home internet access	129 (99.2)
Internet usage	
Daily (or several times a day)	125 (96.9)
Several times a week	2 (1.6)
Once a week	1 (0.8)
Never	1 (0.8)

^a^ There were 4 variables with missing values: gender, 2 participants; translator preferred, 2 participants; education, 2 participants; and material deprivation, 3 participants.

The audiological assessment (reference standard) results for 1 participant were not uploaded to the provincial medical record and thus, were not available for any of the calibration and discrimination analyses against the reference standard. However, their results were included in the test-retest reliability analyses of the index tests. Six participants did not complete every index test: 2 did not complete either of the SHOEBOX tests due to a technical failure, and 4 did not complete the second SHOEBOX test (1 because of a technical failure and 3 who declined). One participant declined to do a second hearWHO test. Because of these exclusions, we report results for 123 to 129 participants, depending on the analysis. There were no adverse events during this study. Ambient noise during administration of the index tests was low (median, 46 dB; IQR, 46-48 dB).

### Reliability

Neither the hearWHO score or grouping nor the SHOEBOX grouping had high test-retest reliability (all κ <0.80), with the SHOEBOX having a κ of 0.64 (95% CI, 0.48-0.79) and hearWHO having a κ of 0.32 (95% CI, 0.18-0.46) (eTable 2 in Supplement 1). We did not measure the test-retest reliability of the reference standard as only 1 assessment is done in clinical practice.

### Calibration and Discrimination for Detecting HL_50_

Flow diagrams for the 6 a priori comparisons of index tests vs HL_50_ are shown in eFigures 1 to 6 in Supplement 1. According to the audiological assessment (reference standard), 21 of 130 participants (16.3%) were identified as having HL_50_, and 93 (72.1%) were identified as having HL_20_ (Table 2). Using the index texts, the number of participants identified as having HL_50_ ranged from 5 (3.9%) with the first SHOEBOX test to 79 (60.8%) with the RHHI-S test. The percentage of participants identified as having HL_20_ was less variable between tests, ranging from 62 (54.0%) with the second SHOEBOX measurement to 100 (76.9%) with the SISA.

**Table 2.** Summary of Hearing Characteristics for All Participants (N = 130)

Hearing test	Participants, No. (%)
**Audiological assessment (reference standard)**	
HL_50_	21 (16.3)
HL_20_	93 (72.1)
No hearing loss^a^	36 (27.9)
**hearWHO (index test)^b^**	
First of 2 measurements	
Score, median (IQR), points	50 (40-50)
Category	
Normal hearing	8 (6.2)
Indeterminate hearing	60 (46.2)
Hearing loss	62 (47.7)
Second of 2 measurements	
Score, median (IQR), points	50 (40-50)
Category	
Normal hearing	11 (8.5)
Indeterminate hearing	73 (56.6)
Hearing loss	45 (34.9)
Definite HL_hearWHO_	
Based on first measurement	62 (47.7)
Based on second measurement	45 (34.9)
Possible HL_hearWHO_	NA
**SHOEBOX (index test)^c^**	
First of 2 measurements	
Category	
Good hearing	52 (40.6)
Hearing loss	71 (55.5)
Significant hearing loss	5 (3.9)
Second of 2 measurements	
Category	
Good hearing	57 (46.0)
Hearing loss	62 (50.0)
Significant hearing loss	5 (4.0)
Definite HL_SHOEBOX_	
Based on first measurement	5 (3.9)
Based on second measurement	5 (4.0)
Possible HL_SHOEBOX_	
Based on first measurement	76 (59.4)
Based on second measurement	67 (54.0)
**SISA (index test)^d^**	
Answers	
No, I always hear everything	30 (23.1)
Yes, sometimes I do not hear what is being said	87 (66.9)
Yes, I regularly do not hear what is being said	13 (10.0)
Yes, I almost never hear what is being said	0
Definite HL_SISA_	100 (76.9)
Possible HL_SISA_	13 (10.0)
**RHHI-S (index test)^e^**	
Score, median (IQR), points	6 (2-14)
Definite HL_RHHI-S_	79 (60.8)
Possible HL_RHHI-S_	NA

Abbreviations: HL_20_, hearing threshold of 20 dB hearing level or greater; HL_50_, hearing threshold of 50 dB hearing level or greater; NA, not applicable; RHHI-S, Revised Hearing Handicap Inventory–Screening; SISA, Single-Item Self-Assessment.

^a^ No hearing loss considered a hearing threshold of less than 20 dB hearing level.

^b^ Definite HL_hearWHO_ defined as less than 50 points on a scale of 0 to 100 points.

^c^ Definite HL_SHOEBOX_ is a SHOEBOX result of significant hearing loss; possible HL_SHOEBOX _is a SHOEBOX result of definite hearing loss or hearing loss.

^d^ The SISA asks 1 question: “Do you have difficulty with your hearing?” Definite HL_SISA_ is an answer of yes, almost never, or yes, regularly; possible HL_SISA_ is any answer except no.

^e^ The RHHI-S is a 10-item questionnaire with a total possible score ranging from 0 to 40 points. Definite HL_RHHI-S_ is a score of at least 6 points.

Table 3 shows the calibration and discrimination statistics for the reference standard of HL_50_ for all the a priori and exploratory index tests. The κ values were less than 0.80, ranging from −0.01 (95% CI, −0.17 to 0.16) for definite HL_SISA_ to 0.38 (95% CI, 0.24-0.51) for the second definite HL_SHOEBOX_ result.

**Table 3.** Calibration and Discrimination of Index Tests vs HL_50_^a^

Index test	No. of participants	κ	C statistic	Sensitivity	Specificity	PPV	NPV
**hearWHO^b^**							
Definite HL_hearWHO_ based on first measurement^c^	129	0.16 (0.03 to 0.29)	0.64 (0.53 to 0.75)	0.71 (0.48 to 0.89)	0.57 (0.47 to 0.66)	0.24 (0.14 to 0.37)	0.91 (0.82 to 0.97)
Definite HL_hearWHO_ based on second measurement^c^	128	0.26 (0.11 to 0.41)	0.69 (0.58 to 0.80)	0.67 (0.43 to 0.85)	0.71 (0.62 to 0.79)	0.31 (0.18 to 0.47)	0.92 (0.83 to 0.97)
**SHOEBOX^d^**							
Definite HL_SHOEBOX_ based on first measurement^c^	127	0.26 (0.13 to 0.39)	0.59 (0.50 to 0.68)	0.19 (0.05 to 0.42)	0.99 (0.95 to 1.00)	0.80 (0.28 to 1.00)	0.86 (0.79 to 0.92)
Definite HL_SHOEBOX_ based on second measurement^c^	123	0.38 (0.24 to 0.51)	0.63 (0.53 to 0.73)	0.26 (0.09 to 0.51)	1.00 (0.97 to 1.00)	1.00 (0.48 to 1.00)	0.88 (0.81 to 0.93)
Possible HL_SHOEBOX_ based on first measurement	127	0.21 (0.10 to 0.32)	0.71 (0.65 to 0.78)	0.95 (0.76 to 1.00)	0.47 (0.37 to 0.57)	0.26 (0.17 to 0.38)	0.98 (0.90 to 1.00)
Possible HL_SHOEBOX_ based on second measurement	123	0.26 (0.15 to 0.38)	0.77 (0.72 to 0.82)	1.00 (0.82 to 1.00)	0.54 (0.44 to 0.64)	0.28 (0.18 to 0.41)	1.00 (0.94 to 1.00)
**SISA^e^**							
Definite HL_SISA_^c^	129	−0.01 (−0.17 to 0.16)	0.50 (0.43 to 0.57)	0.10 (0.01 to 0.30)	0.90 (0.83 to 0.95)	0.15 (0.02 to 0.45)	0.84 (0.76 to 0.90)
Possible HL_SISA_	129	0.07 (−0.01 to 0.14)	0.58 (0.51 to 0.66)	0.91 (0.70 to 0.99)	0.26 (0.18 to 0.35)	0.19 (0.12 to 0.28)	0.93 (0.78 to 0.99)
**RHHI-S^f^**							
Definite HL_RHHI-S_^c^	129	0.08 (−0.02 to 0.19)	0.59 (0.48 to 0.69)	0.76 (0.53 to 0.92)	0.42 (0.32 to 0.52)	0.20 (0.12 to 0.31)	0.90 (0.78 to 0.97)

Abbreviations: HL_50_, hearing threshold of 50 dB hearing level of greater; NPV, negative predictive value; PPV, positive predictive value; RHHI-S, Revised Hearing Handicap Inventory–Screening; SISA, Single-Item Self-Assessment.

^a^ Defined by a pure-tone average of HL_50_ on the reference standard.

^b^ Definite HL_hearWHO_ is less than 50 points on a scale of 0 to 100 points.

^c^ Index test and the reference standard are concordant (ie, the index tests are designed to identify definite rather than possible hearing loss).

^d^ Definite HL_SHOEBOX_ is a SHOEBOX result of significant hearing loss; possible HL_SHOEBOX _is a SHOEBOX result of definite hearing loss or hearing loss.

^e^ The SISA asks 1 question: “Do you have difficulty with your hearing?” Definite HL_SISA_ is an answer of yes, almost never, or yes, regularly; possible HL_SISA _is any answer except no.

^f^ The RHHI-S is a 10-item questionnaire with a total possible score ranging from 0 to 40 points. Definite HL_RHHI-S_ is a score of at least 6 points.

All the C statistics for detecting HL_50_ were less than 0.80. They ranged from 0.50 (95% CI, 0.43-0.57) for definite HL_SISA_ to 0.77 (95% CI, 0.72-0.82) for the second possible HL_SHOEBOX_. The latter had high sensitivity (1.00; 95% CI, 0.82-1.00) and negative predictive value (1.00; 95% CI, 0.94-1.00) but poor specificity (0.54; 95% CI, 0.44-0.64) and positive predictive value (0.28; 95% CI, 0.18-0.41). Sensitivity and specificity for the second measurement of SHOEBOX were 0.26 (95% CI, 0.09-0.51) and 1.00 (95% CI, 0.97-1.00), respectively, and for the second measurement of hearWHO, 0.67 (95% CI, 0.43-0.85) and specificity, 0.71 (95% CI, 0.62-0.79). The RHHI-S sensitivity for HL_50_ was 0.76 (95% CI, 0.53-0.92) and specificity, 0.42 (95% CI, 0.32-0.52). For HL_50_, the SISA sensitivity was 0.10 (95% CI, 0.01-0.30) and specificity, 0.90 (95% CI, 0.83-0.95). The point estimates along the receiver operating characteristic curve for all these comparisons with the reference standard are shown in Figure, A.

**Figure.**
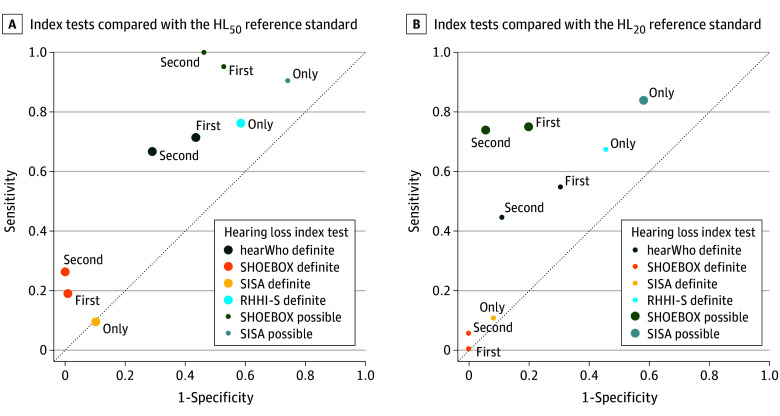
Point Estimates of the Index Tests Compared With the Reference Standard Along the Receiver Operating Characteristic Curve The circles show whether the index test and the reference standard were concordant (both representing more severe or less severe hearing loss). A, Index tests were designed to identify definite rather than possible hearing loss. B, Index tests were designed to identify possible rather than definite hearing loss. The smaller circles show whether the index test and the reference standard were discordant (each targeting a different severity of hearing loss). The markers are labeled to show whether the result came from the first, second, or only measurement for that specific index test. HL_20_ indicates hearing threshold of 20 dB or greater; HL_50_, hearing threshold of 50 dB or greater; RHHI-S, Revised Hearing Handicap Inventory–Screening; SISA, Single-Item Self-Assessment.

### Calibration and Discrimination for Detecting HL_20_

Flow diagrams for the 3 a priori comparisons of index tests vs HL_20_ are shown in eFigures 7 to 9 in Supplement 1. Table 4 shows the calibration and discrimination statistics for HL_20_ for all the a priori and exploratory index tests. In general, the diagnostic performance of the index tests was stronger for detecting HL_20_ than HL_50_.

**Table 4.** Calibration and Discrimination of Index Tests vs HL_20_^a^

Index test	No. of participants	κ	C statistic	Sensitivity	Specificity	PPV	NPV
**hearWHO** ^b^							
Definite HL_hearWHO_ based on first measurement	129	0.19 (0.04 to 0.34)	0.62 (0.53 to 0.71)	0.55 (0.44 to 0.65)	0.69 (0.52 to 0.84)	0.82 (0.71 to 0.91)	0.37 (0.26 to 0.50)
Definite HL_hearWHO_ based on second measurement	128	0.24 (0.11 to 0.37)	0.67 (0.59 to 0.74)	0.45 (0.34 to 0.55)	0.89 (0.74 to 0.97)	0.91 (0.79 to 0.98)	0.39 (0.28 to 0.50)
**SHOEBOX** ^c^							
Definite HL_SHOEBOX_ based on first measurement	127	0.03 (−0.01 to 0.07)	0.53 (0.50 to 0.55)	0.05 (0.02 to 0.12)	1.00 (0.90 to 1.00)	1.00 (0.48 to 1.00)	0.29 (0.21 to 0.38)
Definite HL_SHOEBOX _based on second measurement	123	0.03 (−0.01 to 0.08)	0.53 (0.50 to 0.55)	0.06 (0.02 to 0.13)	1.00 (0.90 to 1.00)	1.00 (0.48 to 1.00)	0.30 (0.22 to 0.39)
Possible HL_SHOEBOX _based on first measurement^d^	127	0.48 (0.31 to 0.65)	0.78 (0.69 to 0.86)	0.75 (0.65 to 0.83)	0.80 (0.63 to 0.92)	0.91 (0.82 to 0.96)	0.55 (0.40 to 0.69)
Possible HL_SHOEBOX _based on second measurement^d^	123	0.58 (0.41 to 0.74)	0.84 (0.78 to 0.90)	0.74 (0.63 to 0.83)	0.94 (0.81 to 0.99)	0.97 (0.90 to 1.00)	0.59 (0.45 to 0.72)
**SISA** ^e^							
Definite HL_SISA_	129	0.01 (−0.05 to 0.08)	0.51 (0.46 to 0.57)	0.11 (0.05 to 0.19)	0.92 (0.78 to 0.98)	0.77 (0.46 to 0.95)	0.28 (0.21 to 0.38)
Possible HL_SISA_^d^	129	0.27 (0.10 to 0.44)	0.63 (0.54 to 0.72)	0.84 (0.75 to 0.91)	0.42 (0.26 to 0.59)	0.79 (0.69 to 0.86)	0.50 (0.31 to 0.69)
**RHHI-S** ^f^							
Definite HL_RHHI-S_	129	0.17 (0.01 to 0.34)	0.60 (0.50 to 0.69)	0.67 (0.56 to 0.76)	0.53 (0.36 to 0.70)	0.79 (0.68 to 0.87)	0.38 (0.25 to 0.53)

Abbreviations: HL_20_, hearing threshold of 20 dB hearing level of greater; NPV, negative predictive value; PPV, positive predictive value; RHHI-S, Revised Hearing Handicap Inventory–Screening; SISA, Single-Item Self-Assessment.

^a^ Defined by a pure-tone average of HL_20_ on the reference standard.

^b^ Definite HL_hearWHO_ is less than 50 points on a scale of 0 to 100 points.

^c^ Definite HL_SHOEBOX_ is a SHOEBOX result of significant hearing loss; possible HL_SHOEBOX _is a SHOEBOX result of definite hearing loss or hearing loss.

^d^ Index test and the reference standard are concordant (ie, the index tests are designed to identify definite rather than possible hearing loss).

^e^ The SISA asks 1 question: “Do you have difficulty with your hearing?” Definite HL_SISA_ is an answer of yes, almost never, or yes, regularly; possible HL_SISA _is any answer except no.

^f^ The RHHI-S is a 10-item questionnaire with a total possible score ranging from 0 to 40 points. Definite HL_RHHI-S_ is a score of at least 6 points.

The κ values for detecting HL_20_ were all less than 0.80, ranging from 0.01 (95% CI, −0.05 to 0.08) for the first definite HL_SHOEBOX_ to 0.58 (95% CI, 0.41-0.74) for the second possible HL_SHOEBOX_. The latter had high specificity (0.94; 95% CI, 0.81-0.99), positive predictive value (0.97; 95% CI, 0.90-1.00), and reasonable sensitivity (0.74; 95% CI, 0.63-0.83) but less optimal negative predictive value (0.59; 95% CI, 0.45-0.72). All but 1 of the C statistics were less than 0.80. They ranged from 0.51 (95% CI, 0.46-0.57) for definite HL_SISA_ to 0.84 (95% CI, 0.78-0.90) for the second possible HL_SHOEBOX_. Figure, B shows the point estimates along the receiver operating characteristic curve for all these comparisons with the reference standard.

### Exploratory Analyses

In exploratory threshold-finding analyses for HL_50_, the first hearWHO score with a threshold of 30 points or less had the highest κ (0.30; 95% CI, 0.16-0.44) and the second hearWHO score with a threshold of 40 points or less had the highest C statistic (0.69; 95% CI, 0.58-0.80) (eTable 3 in Supplement 1). For RHHI-S, a threshold of 25 points or less produced the largest κ (0.02; 95% CI, −0.01 to 0.05) and C statistic (0.53; 95% CI, 0.51-0.55) (eTable 4 in Supplement 1). For RHHI-S, the κ values were almost all negative.

## Discussion

In this prospective diagnostic accuracy study, we compared the validity and reliability of 2 widely recommended mobile hearing loss detection apps with a clinical reference standard based on audiological assessment. The study had 6 key findings. First, both apps had relatively low test-retest reliability. Second, sensitivity and specificity for both apps improved when only the second testing value was considered, which may be due to a learning effect among participants and suggests that administering the test twice may be useful in practice.^22^ Third, sensitivity and specificity of the second hearWHO value to detect HL_50_ were 67% and 71%, respectively, compared with sensitivity and specificity of 26% and 100% for the second SHOEBOX value. Fourth, the SHOEBOX app could be used to identify HL_50_ as well as HL_20_; its sensitivity and specificity were superior for the latter. Fifth, when using the SHOEBOX app and aiming to identify people with HL_50_, selecting a less stringent diagnostic threshold increased sensitivity to 100%, while retaining 54% specificity. Sixth, attempts to improve the sensitivity and specificity of the hearWHO app by selecting an alternative threshold to define HL_50_ did not yield a clear advantage compared with the recommended threshold.

These findings suggest that the suitability of each app may depend on the intended purpose. If the goal is to identify most people who have HL_20_ or greater (a sensitive strategy), then either the hearWHO app or SHOEBOX app could be selected, although specificity may be relatively low. On the other hand, if the goal is to identify people who may have HL_50_ or greater, while also minimizing false-positive results (a specific strategy), it may be most appropriate to use the SHOEBOX app. In either case, it will be important to select the appropriate diagnostic threshold (possible HL_SHOEBOX_ for sensitivity, definite HL_SHOEBOX_ for specificity) to define hearing loss if the SHOEBOX app is used. If neither the hearWHO nor the SHOEBOX app is available, the RHHI-S was found to be moderately sensitive for HL_50_ (though not specific), whereas the slightly less complex SISA was a reasonably specific (but not sensitive) alternative for detecting HL_20_. In settings where audiological assessment is available, an abnormal result for any of the 4 index tests could be used to trigger referral for a comprehensive audiological assessment. In situations where assessment is unavailable or unaffordable, abnormal results might be sufficient to trigger a lower-intensity intervention, such as assistive listening devices or closed captioning.^23^

Previous studies comparing the SHOEBOX app with audiometry in adults have had sample sizes ranging from 25 to 73 participants and typically used a threshold of more than 25 dB HL to define hearing loss.^12,24,25,26^ Some studies used the ear or the frequency of testing as the unit of the analysis rather than the individual. The sensitivity (0.87-1.00) and specificity (0.82-0.96) values reported in these studies tended to be higher than ours perhaps because of these methodological differences. Neither our search nor a recent systematic review^12^ identified studies that compared the SHOEBOX app with the hearWHO app or the SHOEBOX app with an HL_50_ threshold as assessed by audiometry to define hearing loss.

Previous studies evaluating prior iterations of the hearWHO app against reference standard audiometry were of high quality and tended to have larger sample sizes than those evaluating the SHOEBOX app.^10,22,27^ All studies evaluating the hearWHO app were done by the same group and used an average hearing threshold of more than 25 dB HL across 4 frequencies to define hearing loss. Sensitivity in these studies ranged from 0.88 to 0.94 and specificity from 0.77 to 0.88. We did not identify studies using the hearWHO app that evaluated a threshold of HL_50_ as assessed by audiometry to define hearing loss.

The findings from our study have potentially important implications for practice. Hearing loss is common worldwide and is often referred to as an invisible or hidden disability because it lacks visible symptoms and is often disregarded politically and societally.^3^ Reducing barriers to hearing tests may help people identify hearing loss earlier and raise awareness about hearing health. We found that both the hearWHO and SHOEBOX apps are suitable when a sensitive approach is preferred, such as quickly testing a broader range of people to determine those who may warrant further testing. On the other hand, the SHOEBOX app may be more suitable than the hearWHO app if a more-specific strategy is preferred, for example, in settings in which access to audiologists may be especially limited.

### Strengths and Limitations

Our study has important strengths, including its rigorous design, use of an appropriate reference standard, comparison with 2 widely recommended mobile apps for detecting hearing loss, and adherence to internationally recognized reporting standards.

Our study also has several limitations to consider when interpreting the results. First, our sample size of 129 participants meant that our statistical power to draw conclusions about differences between the 2 apps was limited but probably did not influence our main conclusions. Second, all participants had been referred for audiological assessment, and the true prevalence of moderate or greater hearing loss was 16.3%. Therefore, the positive and negative predictive values that we report may vary in populations for which the expected prevalence of hearing loss is lower (eg, the general adult population) or higher (eg, hospitalized older adults). Third, unlike some prior studies,^25,26^ we did not calibrate the tablet and headphones to American National Standards Institute standards before using the SHOEBOX app. However, since the manufacturers did not recommend such calibration, it is uncertain whether not calibrating the tablet and headphones influenced the applicability of our results or conclusions. Finally, we were unable to mask participants to the results of the reference standard test (audiological assessment), which was always done prior to the index tests. Although knowledge of their hearing loss status may have influenced some participants’ performance on the 2 index tests or decision to participate, it should not have influenced comparisons of either index test with the reference standard.

## Conclusions

In this diagnostic accuracy study of 2 hearing detection apps, we found that the hearWHO app and the SHOEBOX app both appeared to be suitable if a sensitive strategy is desired for detecting people who may benefit from definitive audiological assessment, whereas the SHOEBOX app may be preferable if a specific strategy is desired. If neither the hearWHO nor SHOEBOX app is available, simple questionnaires such as the SISA (more sensitive) or RHHI-S (more specific) may also be useful for detecting hearing loss. Given that hearing loss often goes unrecognized in the general population, all these instruments may be helpful for reducing the societal burden of this common condition. Future work should explore the use of these apps in practice, such as informing the decision to refer for audiological assessment.

## Notes

Supplement 1. **eTable 1.** Index Tests for Hearing Loss **eTable 2.** Test-Rest Reliability **eTable 3.** Threshold Finding: hearWHO vs Reference Standard **eTable 4.** Threshold Finding: RHHI-S vs Reference Standard **eFigure 1.** Participant Flow Diagram: First Measurement of hearWHO vs HL_50_ on Reference Standard **eFigure 2.** Participant Flow Diagram: Second Measurement of hearWHO vs HL_50_ on Reference Standard **eFigure 3.** Participant Flow Diagram: First Measurement of SHOEBOX vs HL_50_ on Reference Standard **eFigure 4.** Participant Flow Diagram: Second Measurement of SHOEBOX vs HL_50_ on Reference Standard **eFigure 5.** Participant Flow Diagram: SISA vs HL_50_ on Reference Standard **eFigure 6.** Participant Flow Diagram: RHHI-S vs HL_50_ on Reference Standard **eFigure 7.** Participant Flow Diagram: First Measurement of SHOEBOX vs HL_20_ on Reference Standard **eFigure 8.** Participant Flow Diagram: Second Measurement of SHOEBOX vs HL_20_ on Reference Standard **eFigure 9.** Participant Flow Diagram: SISA vs HL_20_ on Reference Standard

Supplement 2.
Data Sharing Statement
