# Supplementary material for: Performance of Hearing Test Software Applications to Detect Hearing Loss
Source: JAMA Netw Open. 2025 Mar 27;8(3):e252166. doi: 10.1001/jamanetworkopen.2025.2166 (PMC11950885; doi:10.1001/jamanetworkopen.2025.2166)
Supplement: Supplement 1. — eTable 1. Index Tests for Hearing Loss eTable 2. Test-Rest Reliability eTable 3. Threshold Finding: hearWHO vs Reference Standard eTable 4. Threshold Finding: RHHI-S vs Reference Standard eFigure 1. Participant Flow Diagram: First Measurement of hearWHO vs HL50 on Reference Standard eFigure 2. Participant Flow Diagram: Second Measurement of hearWHO vs HL50 on Reference Standard eFigure 3. Participant Flow Diagram: First Measurement of SHOEBOX vs HL50 on Reference Standard eFigure 4. Participant Flow Diagram: Second Measurement of SHOEBOX vs HL50 on Reference Standard eFigure 5. Participant Flow Diagram: SISA vs HL50 on Reference Standard eFigure 6. Participant Flow Diagram: RHHI-S vs HL50 on Reference Standard eFigure 7. Participant Flow Diagram: First Measurement of SHOEBOX vs HL20 on Reference Standard eFigure 8. Participant Flow Diagram: Second Measurement of SHOEBOX vs HL20 on Reference Standard eFigure 9. Participant Flow Diagram: SISA vs HL20 on Reference Standard [file jamanetwopen-e252166-s001.pdf]

Supplemental Online Content

Lunney M, Wiebe N, Howarth T, et al. Performance of mobile applications to detect hearing loss. *JAMA Netw Open*. 2025;3(3):e252166. doi:10.1001/jamanetworkopen.2025.2166

eTable 1. Index Tests for Hearing Loss

eTable 2. Test-Rest Reliability

eTable 3. Threshold Finding: hearWHO vs Reference Standard

eTable 4. Threshold Finding: RHHI-S vs Reference Standard

eFigure 1. Participant Flow Diagram: First Measurement of hearWHO vs HL_{50} on Reference Standard

eFigure 2. Participant Flow Diagram: Second Measurement of hearWHO vs HL_{50} on Reference Standard

eFigure 3. Participant Flow Diagram: First Measurement of SHOEBOX vs HL_{50} on Reference Standard

eFigure 4. Participant Flow Diagram: Second Measurement of SHOEBOX vs HL_{50} on Reference Standard

eFigure 5. Participant Flow Diagram: SISA vs HL_{50} on Reference Standard

eFigure 6. Participant Flow Diagram: RHHI-S vs HL_{50} on Reference Standard

eFigure 7. Participant Flow Diagram: First Measurement of SHOEBOX vs HL_{20} on Reference Standard

eFigure 8. Participant Flow Diagram: Second Measurement of SHOEBOX vs HL_{20} on Reference Standard

eFigure 9. Participant Flow Diagram: SISA vs HL_{20} on Reference Standard

This supplemental material has been provided by the authors to give readers additional information about their work.

© 2025 Lunney M et al. *JAMA Network Open*.

eTable 1. Index Tests for Hearing Loss

Test	Description	Details	Definitions	
			Definite HL	Possible HL
hearWHO	A smartphone app determines how loud a sound needs to be in order to be heard by the participant. Ears are tested together.	The returned score ranges from 0-100. It categorizes the participant into 3 groups: normal hearing (score >75), indeterminate hearing (score 50-75), and hearing loss (score <50).	Definite $HL_{hearWHO}$ = Score <50	-
SHOEBOX	An online test (shoebox.md) that combines self-reported measures (3 questions) with pure-tone technology (6 threshold stimuli). Ears are tested individually.	The online test does not return a score although the pictogram suggests one. It categorizes the participants into 3 groups: good hearing, hearing loss, and significant hearing loss. Take the best of both ears.	Definite $HL_{SHOEBOX}$ = "Significant HL"	Possible $HL_{SHOEBOX}$ = "HL" or "Significant HL"
Single-item self-assessment tool[1]	The single-item question is designed to assess overall self-reported HL. Ears are tested together.	Do you have any difficulty with your hearing?<br>-No, I hear everything (i.e.,No)<br>-Yes, sometimes I do not hear what is being said (i.e., Sometimes)<br>-Yes, I regularly do not hear what is being said (i.e., Often)<br>-Yes, I almost never hear what is being said (i.e., Almost always)	Definite HL_{SISA} = "Yes, almost always …" or "Yes, regularly …"	Possible HL_{SISA} – "Yes, almost always …", "Yes, regularly …", or "Yes, sometimes …"
Revised hearing handicap inventory screening[2]	This 10-item survey is designed to measure self-perceived hearing disability. Ears are tested together.	An answer of 'Yes' is 4 points, 'Sometimes' is 2 points, and 'No' is 0 points, meaning that the higher the score, the greater the HL. The total possible score ranges from 0 to 40.	Definite HL_{RHHI-S} = Score ≥6	-

HL hearing loss, PTA pure tone average, RHHI-S Revised hearing handicap inventory screening
No hearing aids were permitted during these tests.

If an index test had only one category that referred to an abnormal result, we described that result as "definite HL". For the two index tests that had two categories that referred to an abnormal result (i.e. more severe and less severe abnormality; SHOEBOX; SISA), we described the more severe abnormality as "definite HL" and the less severe abnormality as "possible HL".

1. Oosterloo BC, Homans NC, Baatenburg de Jong RJ, Ikram MA, Nagtegaal AP, Goedegebure A. Assessing hearing loss in older adults with a single question and person characteristics; Comparison with pure tone audiometry in the Rotterdam Study. PLoS One. 2020;15(1):e0228349
2. Cassarly C, Matthews LJ, Simpson AN, Dubno JR. The Revised Hearing Handicap Inventory and Screening Tool Based on Psychometric Reevaluation of the Hearing Handicap Inventories for the Elderly and Adults. Ear Hear. 2020;41:95-105.

© 2025 Lunney M et al. *JAMA Network Open*.

eTable 2. Test-Retest Reliability

Tool	N	Agreement	Kappa
hearWHO score (continuous: 0-100)	129	0.47	0.21[1] (0.10,0.32)
hearWHO (categorical: 3 groups)	129	0.61	0.32 (0.18,0.46)
SHOEBOX group (categorical: 3 groups)	124	0.81	0.64 (0.48,0.79)

[1]Correlation is 0.53.

© 2025 Lunney M et al. *JAMA Network Open*.

eTable 3. Threshold Finding: hearWHO vs Reference Standard

Threshold	N	Kappa	C-statistic	Sensitivity	Specificity	PPV	NPV
HL_{50}							
**hearWHO** score from first measurement							
≤30	129	0.30 (0.16,0.44)	0.61 (0.52,0.70)	0.24 (0.08,0.47)	0.98 (0.94,1.00)	0.71 (0.29,0.96)	0.87 (0.80,0.92)
≤40		0.16 (0.03,0.29)	0.64 (0.53,0.75)	0.71 (0.48,0.89)	0.57 (0.47,0.66)	0.24 (0.14,0.37)	0.91 (0.82,0.97)
≤50		0.03 (-0.04,0.09)	0.54 (0.46,0.61)	0.91 (0.70,0.99)	0.17 (0.10,0.25)	0.17 (0.11,0.26)	0.90 (0.68,0.99)
≤60		0.02 (-0.03,0.07)	0.53 (0.47,0.58)	0.95 (0.76,1.00)	0.10 (0.05,0.18)	0.17 (0.11,0.25)	0.92 (0.62,1.00)
≤70		0.03 (-0.01,0.06)	0.54 (0.51,0.56)	1.00 (0.84,1.00)	0.07 (0.03,0.14)	0.17 (0.11,0.25)	1.00 (0.63,1.00)
**hearWHO** score from second measurement							
≤30	129	0.08 (0.01,0.14)	0.52 (0.48,0.57)	0.05 (0.01,0.24)	1.00 (0.97,1.00)	1.00 (0.03,1.00)	0.84 (0.77,0.90)
≤40		0.26 (0.11,0.41)	0.69 (0.58,0.80)	0.67 (0.43,0.85)	0.71 (0.62,0.80)	0.31 (0.18,0.47)	0.92 (0.84,0.97)
≤50		0.03 (-0.05,0.10)	0.54 (0.45,0.62)	0.86 (0.64,0.97)	0.21 (0.14,0.30)	0.18 (0.11,0.26)	0.89 (0.70,0.98)
≤60		0.02 (-0.04,0.08)	0.53 (0.45,0.60)	0.91 (0.70,0.99)	0.15 (0.09,0.23)	0.17 (0.11,0.25)	0.89 (0.65,0.99)
≤70		0.04 (-0.01,0.08)	0.55 (0.52,0.58)	1.00 (0.84,1.00)	0.10 (0.05,0.18)	0.18 (0.11,0.26)	1.00 (0.72,1.00)
HL_{20}							
**hearWHO** score from first measurement							
≤30	129	0.04 (-0.01,0.09)	0.54 (0.51,0.56)	0.08 (0.03,0.15)	1.00 (0.90,1.00)	1.00 (0.59,1.00)	0.30 (0.22,0.38)
≤40		0.19 (0.04,0.34)	0.62 (0.53,0.71)	0.55 (0.44,0.65)	0.69 (0.52,0.84)	0.82 (0.71,0.91)	0.37 (0.26,0.50)
≤50		0.24 (0.08,0.40)	0.60 (0.52,0.69)	0.90 (0.82,0.96)	0.31 (0.16,0.48)	0.77 (0.68,0.85)	0.55 (0.32,0.77)
≤60		0.13 (-0.01,0.27)	0.55 (0.48,0.62)	0.94 (0.87,0.98)	0.17 (0.06,0.33)	0.74 (0.66,0.82)	0.50 (0.21,0.79)
≤70		0.04 (-0.08,0.16)	0.51 (0.46,0.57)	0.95 (0.88,0.98)	0.08 (0.02,0.23)	0.73 (0.64,0.80)	0.38 (0.09,0.76)
**hearWHO** score from second measurement							
≤30	129	0.01 (-0.01,0.02)	0.51 (0.49,0.52)	0.01 (0.00,0.06)	1.00 (0.90,1.00)	1.00 (0.03,1.00)	0.28 (0.21,0.37)
≤40		0.23 (0.10,0.36)	0.66 (0.59,0.74)	0.44 (0.34,0.55)	0.89 (0.74,0.97)	0.91 (0.79,0.98)	0.38 (0.28,0.49)
≤50		0.33 (0.16,0.49)	0.65 (0.56,0.74)	0.88 (0.80,0.94)	0.42 (0.26,0.59)	0.80 (0.71,0.87)	0.58 (0.37,0.77)
≤60		0.32 (0.16,0.47)	0.63 (0.55,0.72)	0.94 (0.87,0.98)	0.33 (0.19,0.51)	0.78 (0.70,0.86)	0.67 (0.41,0.87)
≤70		0.19 (0.06,0.33)	0.58 (0.51,0.64)	0.96 (0.89,0.99)	0.19 (0.08,0.36)	0.75 (0.67,0.83)	0.64 (0.31,0.89)

HL hearing loss, NPV negative predictive value, PPV positive predictive value, PTA pure tone average

The definitions for definite HL and definite or possible HL for the reference standard are given in eTable 1.

© 2025 Lunney M et al. *JAMA Network Open*.

eTable 4. Threshold Finding: RHHI-S vs Reference Standard

Threshold	N	Kappa	C-statistic	Sensitivity	Specificity	PPV	NPV
HL_{50}							
**RHHI-S** score							
≤5	129	-0.12 (-0.26,0.03)	0.41 (0.31,0.52)	0.24 (0.08,0.47)	0.58 (0.49,0.68)	0.10 (0.03,0.22)	0.80 (0.69,0.88)
≤10		-0.14 (-0.23,-0.04)	0.34 (0.23,0.46)	0.43 (0.22,0.66)	0.26 (0.18,0.35)	0.10 (0.05,0.18)	0.70 (0.54,0.83)
≤15		-0.09 (-0.16,-0.01)	0.39 (0.28,0.50)	0.62 (0.38,0.82)	0.16 (0.09,0.24)	0.13 (0.07,0.20)	0.68 (0.47,0.85)
≤20		-0.01 (-0.05,0.03)	0.48 (0.42,0.55)	0.91 (0.70,0.99)	0.07 (0.03,0.13)	0.16 (0.10,0.24)	0.78 (0.40,0.97)
≤25		0.02 (-0.01,0.05)	0.53 (0.51,0.55)	1.00 (0.84,1.00)	0.06 (0.02,0.12)	0.17 (0.11,0.25)	1.00 (0.54,1.00)
HL_{20}							
**RHHI-S** score							
≤5	129	-0.14 (-0.28,-0.01)	0.40 (0.31,0.50)	0.33 (0.24,0.44)	0.47 (0.30,0.65)	0.62 (0.47,0.75)	0.22 (0.13,0.32)
≤10		-0.16 (-0.33,0.02)	0.42 (0.34,0.50)	0.65 (0.54,0.74)	0.19 (0.08,0.36)	0.67 (0.57,0.77)	0.18 (0.07,0.33)
≤15		-0.17 (-0.34,0.00)	0.42 (0.36,0.49)	0.76 (0.66,0.85)	0.08 (0.02,0.23)	0.68 (0.58,0.77)	0.12 (0.03,0.31)
≤20		-0.03 (-0.15,0.10)	0.49 (0.44,0.54)	0.93 (0.85,0.97)	0.06 (0.01,0.19)	0.72 (0.63,0.80)	0.22 (0.03,0.60)
≤25		-0.03 (-0.14,0.07)	0.49 (0.45,0.52)	0.95 (0.88,0.98)	0.03 (0.00,0.15)	0.72 (0.63,0.79)	0.17 (0.00,0.64)

HL hearing loss, NPV negative predictive value, PPV positive predictive value, PTA pure tone average, RHHI-S Revised Hearing Handicap Inventory – Screening

The definitions for definite HL and definite or possible HL for the reference standard are given in eTable 1.

© 2025 Lunney M et al. *JAMA Network Open*.

eFigure 1. Participant Flow Diagram: First Measurement of hearWHO vs HL_{50} on Reference Standard

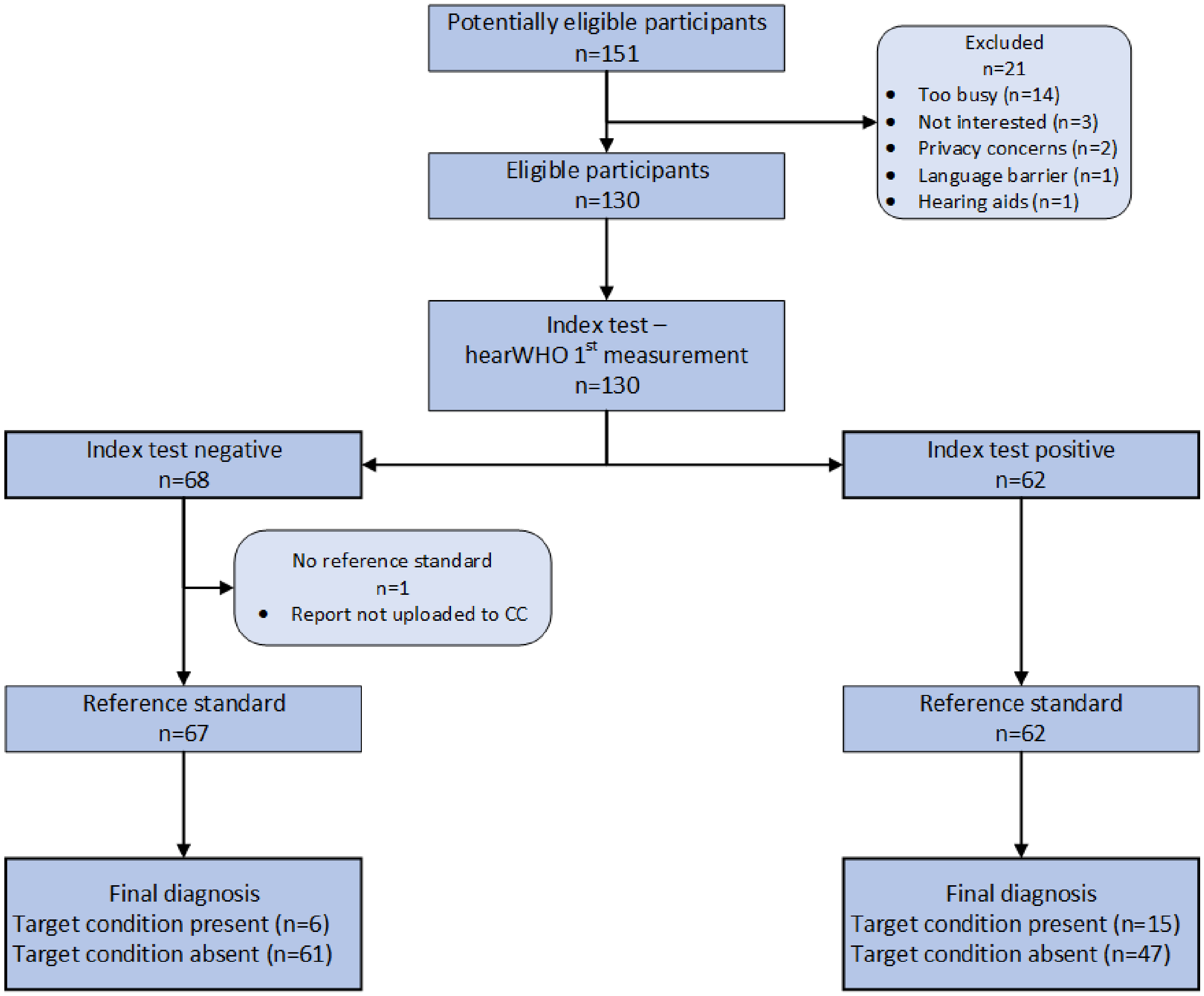

CC ConnectCare, HL hearing loss

© 2025 Lunney M et al. *JAMA Network Open*.

eFigure 2. Participant Flow Diagram: Second Measurement of hearWHO vs HL_{50} on Reference Standard

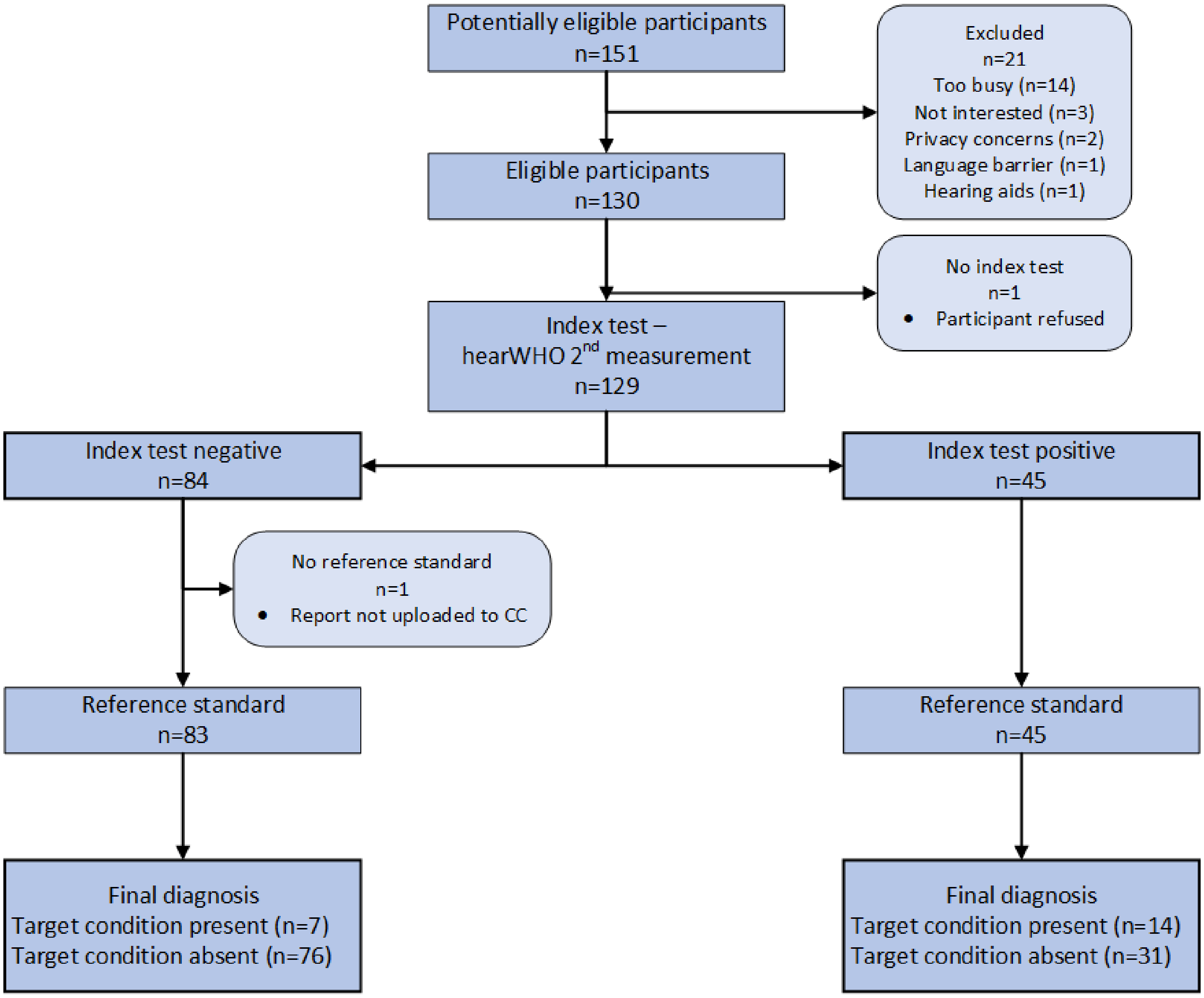

CC ConnectCare, HL hearing loss

© 2025 Lunney M et al. *JAMA Network Open*.

eFigure 3. Participant Flow Diagram: First Measurement of SHOEBOX vs HL_{50} on Reference Standard

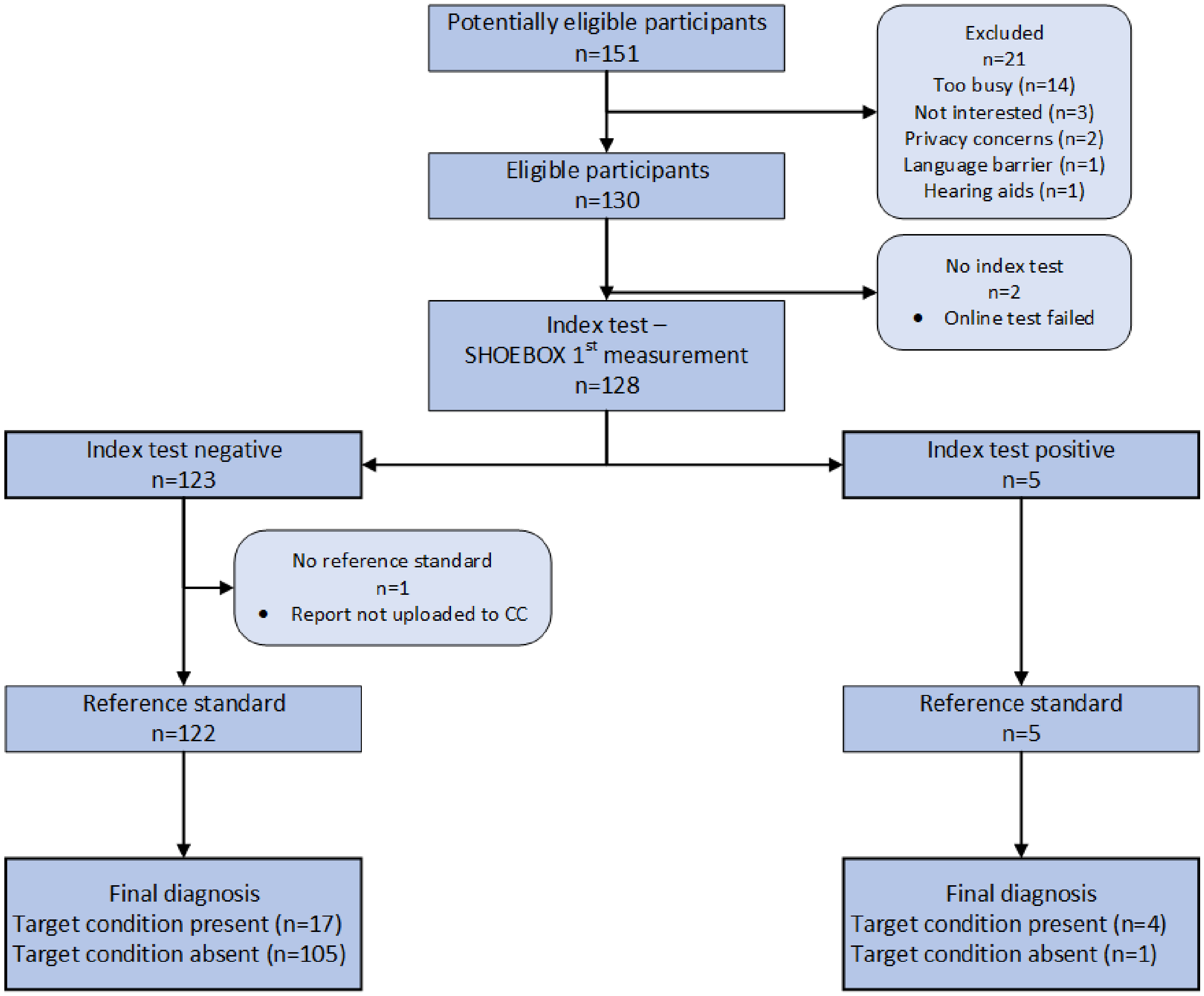

CC ConnectCare, HL hearing loss

© 2025 Lunney M et al. *JAMA Network Open*.

eFigure 4. Participant Flow Diagram: Second Measurement of SHOEBOX vs HL_{50} on Reference Standard

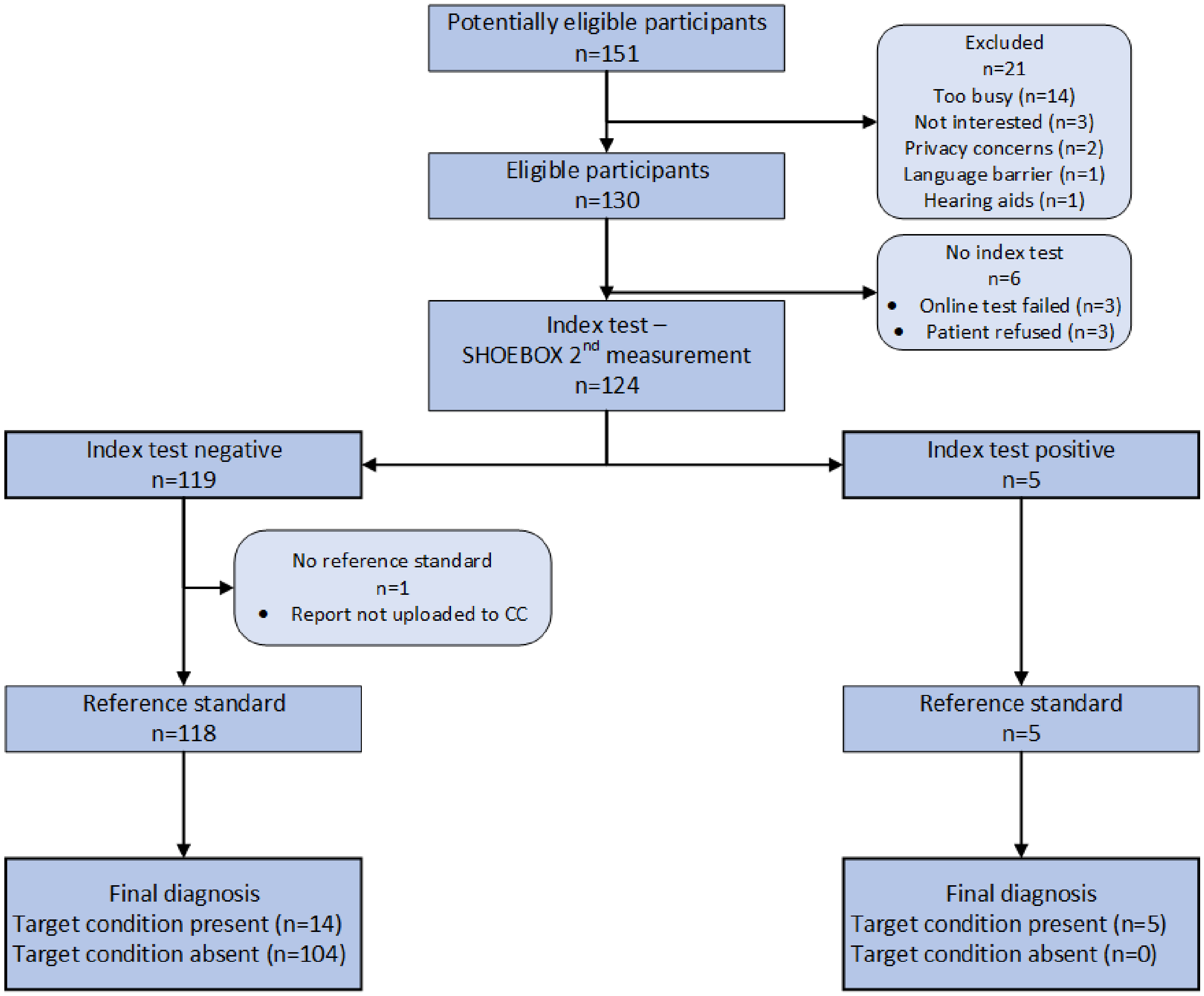

CC ConnectCare, HL hearing loss

© 2025 Lunney M et al. *JAMA Network Open*.

eFigure 5. Participant Flow Diagram: SISA vs HL_{50} on Reference Standard

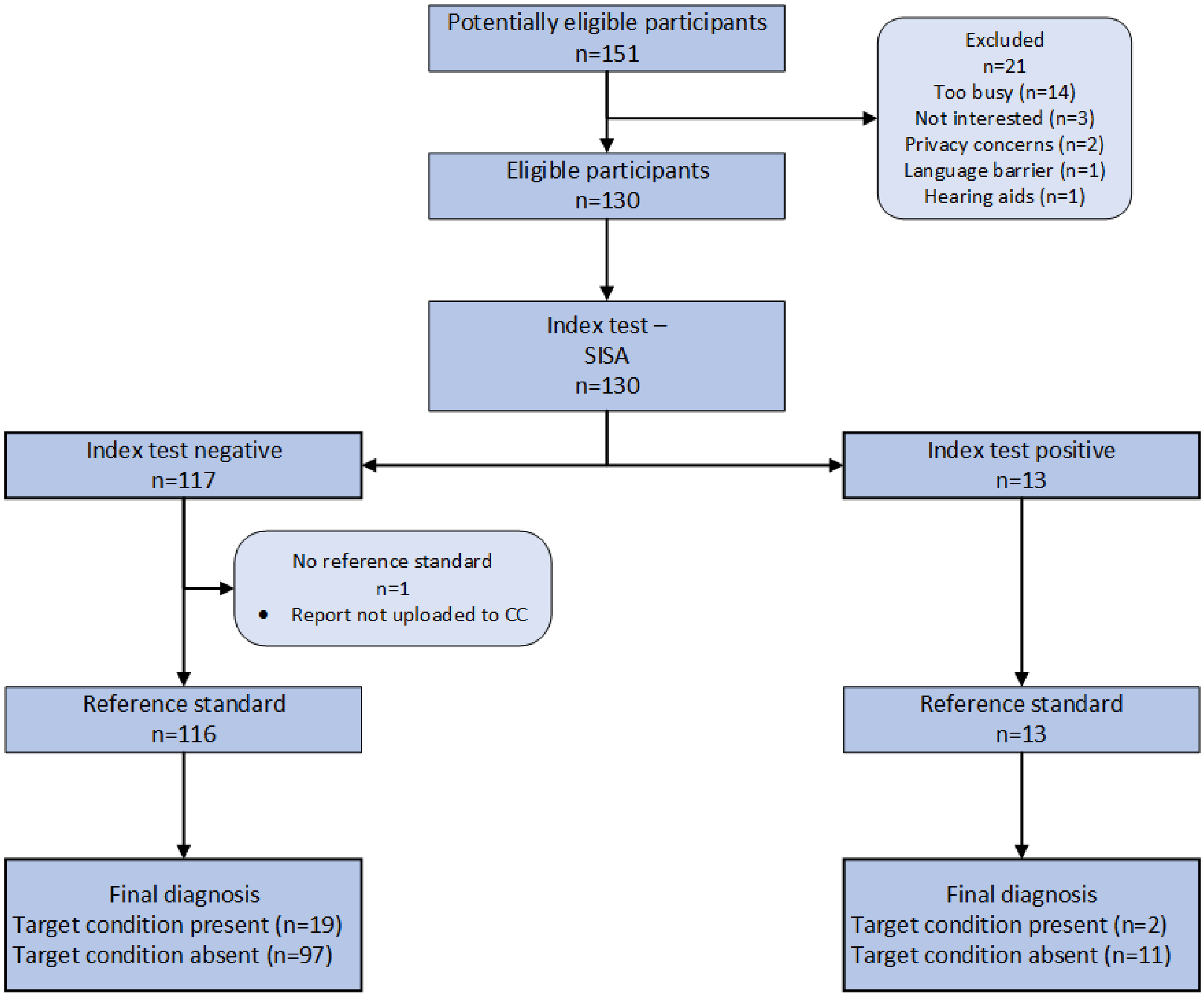

CC ConnectCare, HL hearing loss, SISA single-item self-assessment

© 2025 Lunney M et al. *JAMA Network Open*.

eFigure 6. Participant Flow Diagram: RHHI-S vs HL_{50} on Reference Standard

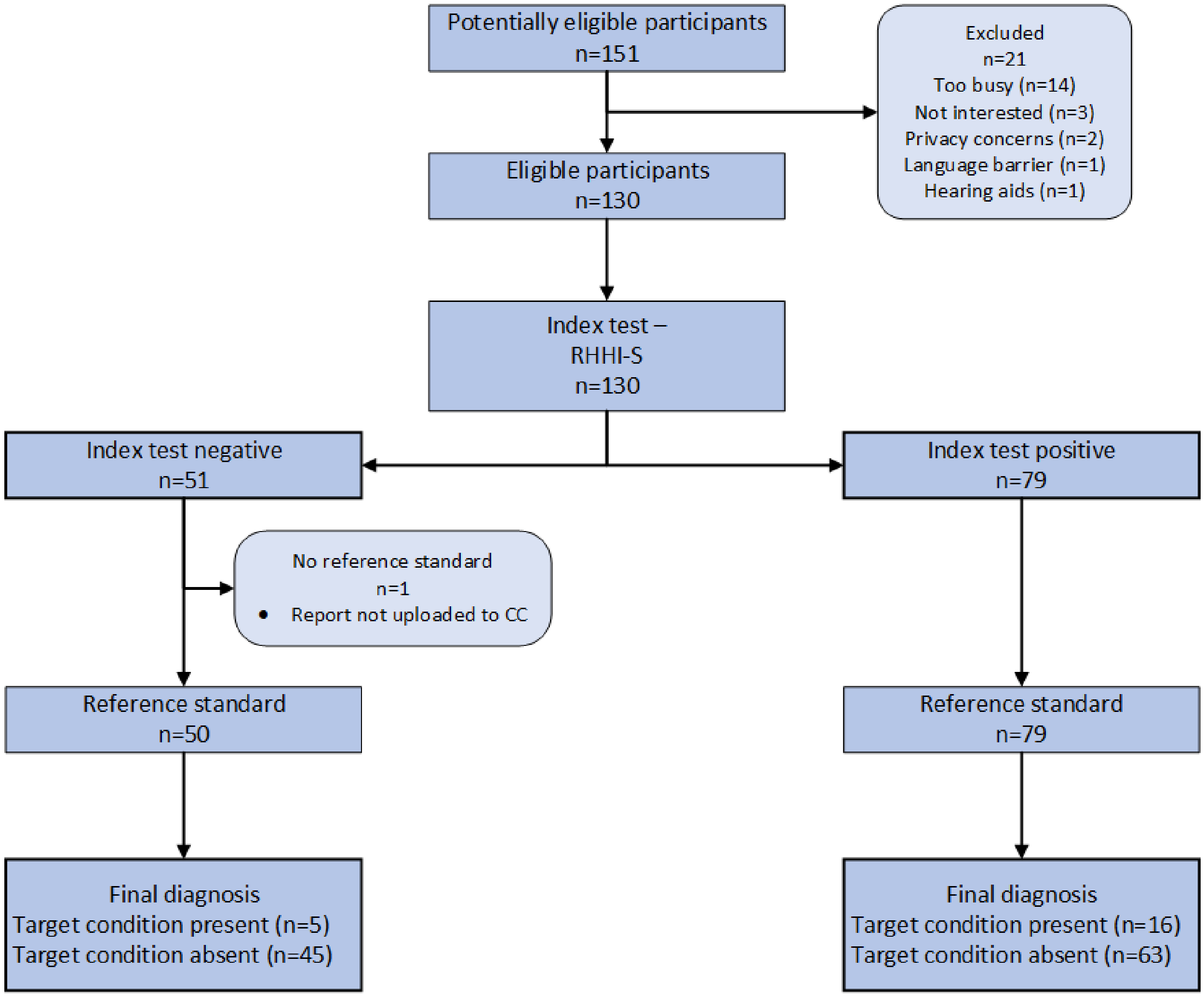

CC ConnectCare, HL hearing loss, RHHI-S Revised Hearing Handicap Inventory Screening

© 2025 Lunney M et al. *JAMA Network Open*.

eFigure 7. Participant Flow Diagram: First Measurement of SHOEBOX vs HL_{20} on Reference Standard

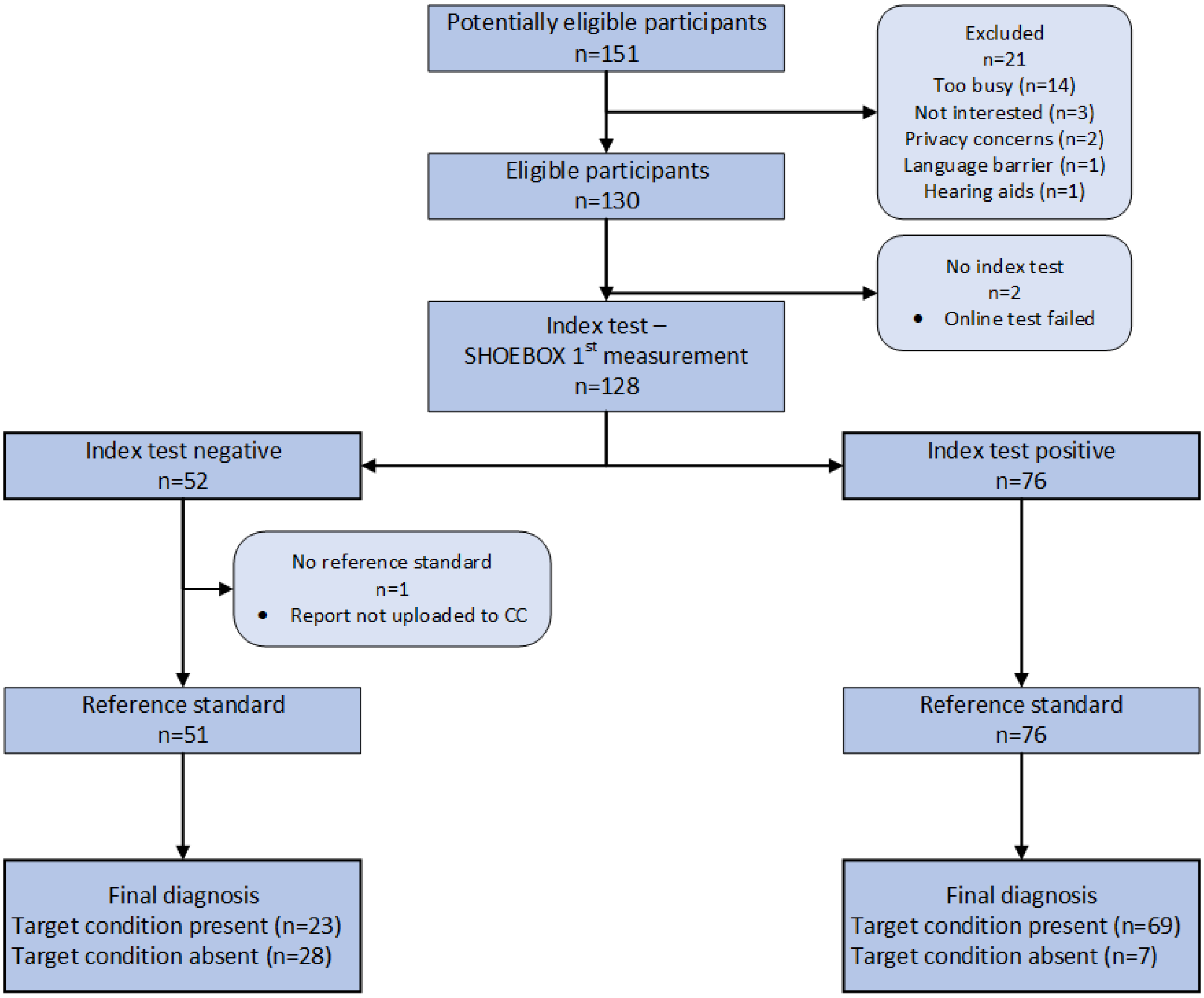

CC ConnectCare, HL hearing loss

© 2025 Lunney M et al. *JAMA Network Open*.

eFigure 8. Participant Flow Diagram: Second Measurement of SHOEBOX vs HL_{20} on Reference Standard

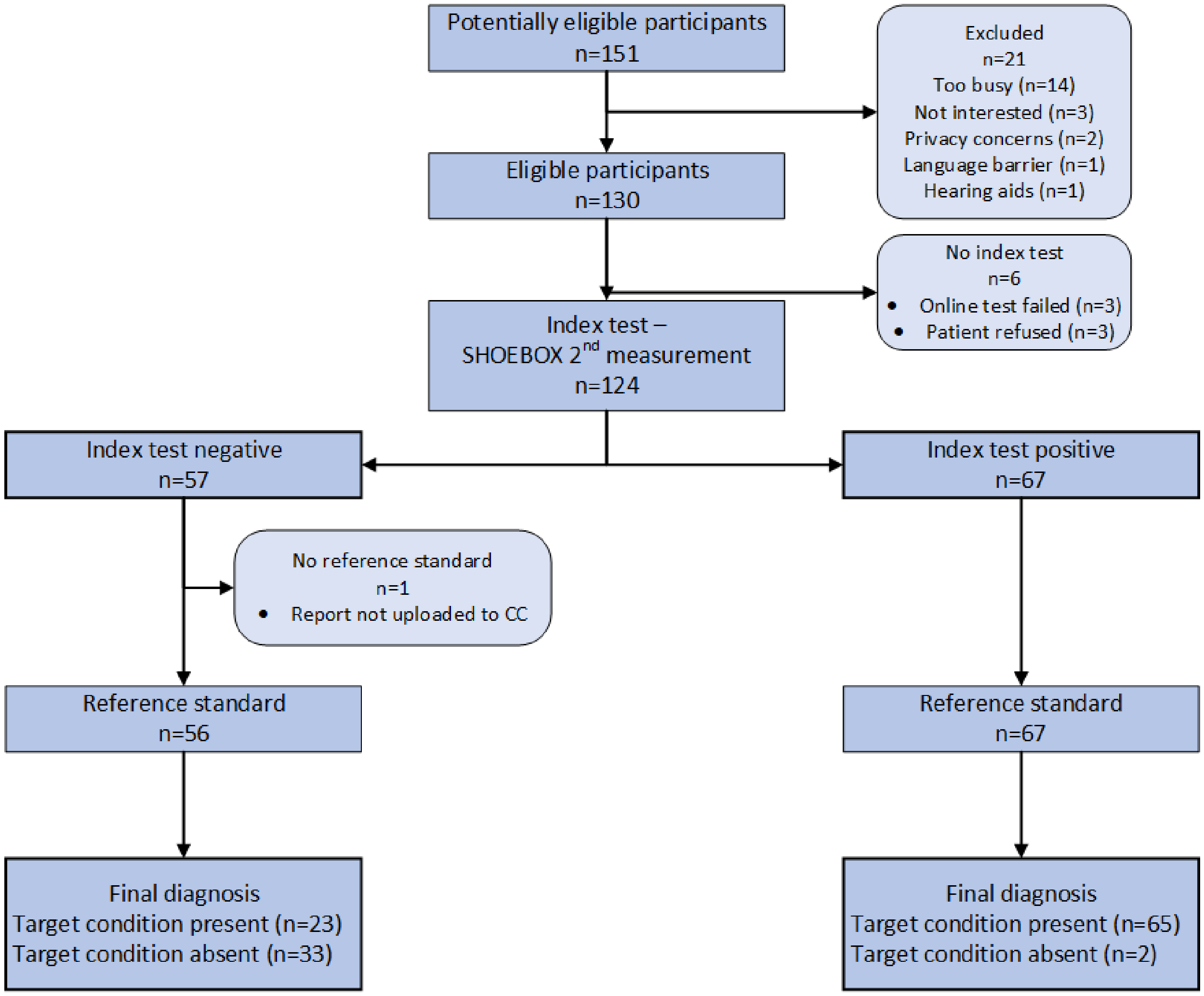

CC ConnectCare, HL hearing loss

© 2025 Lunney M et al. *JAMA Network Open*.

eFigure 9. Participant Flow Diagram: SISA vs HL_{20} on reference standard

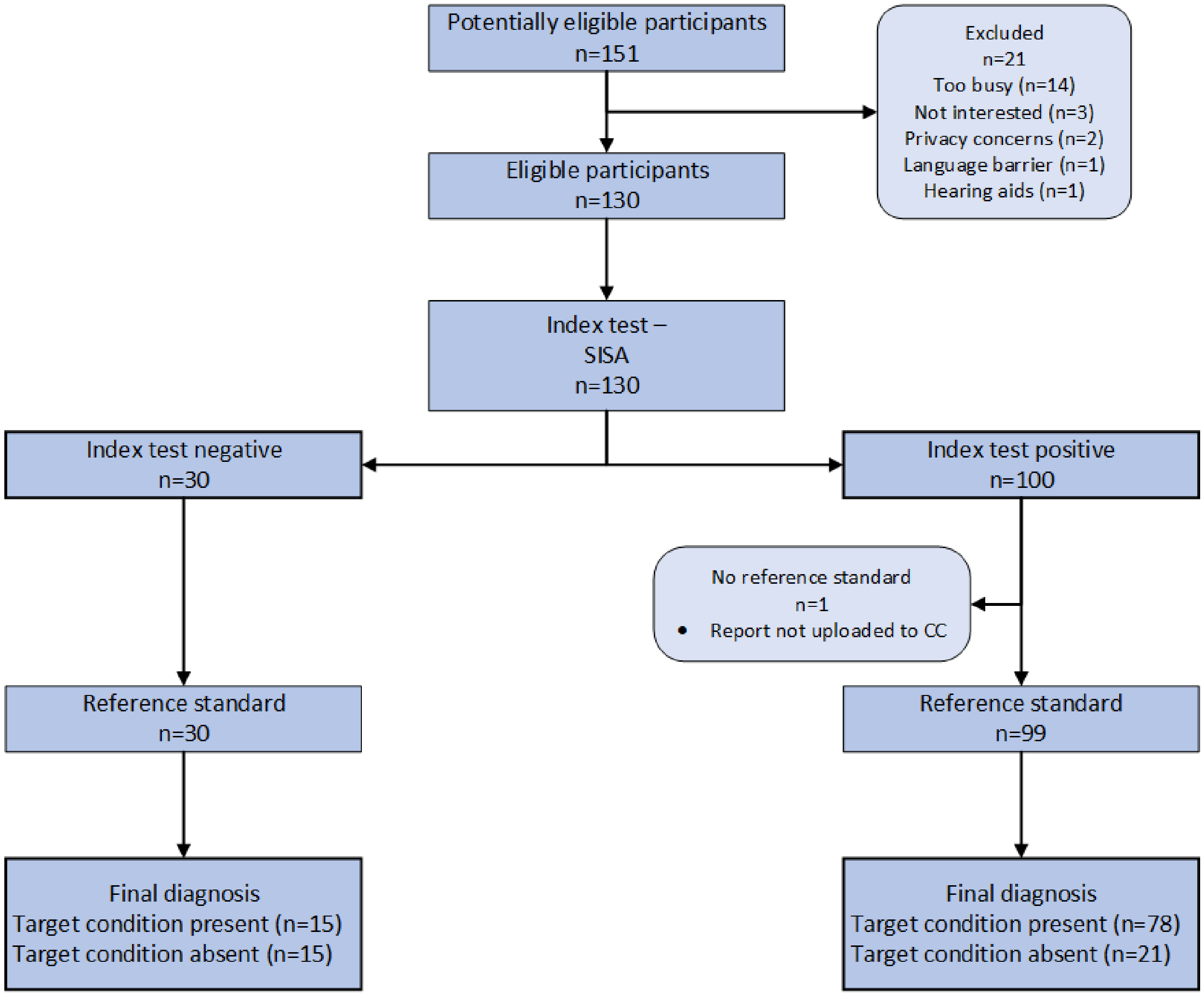

CC ConnectCare, HL hearing loss, SISA single-item self-assessment

© 2025 Lunney M et al. *JAMA Network Open*.
