# Supplementary material for: Performance of Hearing Test Software Applications to Detect Hearing Loss
Source: JAMA Netw Open. 2025 Mar 27;8(3):e252166. doi: 10.1001/jamanetworkopen.2025.2166 (PMC11950885; doi:10.1001/jamanetworkopen.2025.2166)
Supplement: Supplement 2. — Data Sharing Statement [file jamanetwopen-e252166-s002.pdf]

Data Sharing Statement

Lunney. Performance of Mobile Applications to Detect Hearing Loss. *JAMA Netw Open*. Published March 27, 2025. doi:10.1001/jamanetworkopen.2025.2166

Data
Data available: No

Additional Information
Explanation for why data not available: The data are not publicly available due to national data protection laws and restrictions imposed by relevant research ethics boards to ensure data privacy of the study participants. However, the data that support the findings of this study are available upon reasonable request from the Research Manager for the Kidney Health Research Group, Natasha Wiebe.
